# The interactive effects between far-red light and temperature on lettuce growth and morphology diminish at high light intensity

**DOI:** 10.3389/fpls.2024.1497672

**Published:** 2024-12-02

**Authors:** Sang Jun Jeong, Qianwen Zhang, Genhua Niu, Shuyang Zhen

**Affiliations:** ^1^ Department of Horticultural Sciences, Texas A&M University, College Station, TX, United States; ^2^ Texas A&M AgriLife Research and Extension Center at Dallas, Dallas, TX, United States; ^3^ Truck Crops Branch Experiment Station, Mississippi State University, Crystal Springs, MS, United States

**Keywords:** indoor farming, photon capture, phytochrome photoequilibrium, plant yield, antioxidant capacity

## Abstract

Phytochromes (PHYs) play a dual role in sensing light spectral quality and temperature. PHYs can interconvert between the active P_fr_ form and inactive P_r_ form upon absorption of red (R) and far-red (FR) light (Photoconversion). In addition, active P_fr_ can be converted to inactive P_r_ in a temperature-dependent manner (Thermal Reversion). Recent studies have shown that FR light and temperature can interactively affect plant growth and morphology through co-regulating phytochrome activities. These studies were primarily conducted under relatively low light intensities. As light intensity increases, the impact of thermal reversion on phytochrome dynamics decreases. However, the light intensity dependency of the interactive effects between FR light and temperature on plant growth and morphology has not been characterized. In this study, lettuce (*Lactuca sativa* L.) ‘Rex’ was grown under two total photon flux densities (TPFD; 400-800 nm) (150 and 300 μmol m^-2^ s^-1^) x three temperatures (20, 24, and 28°C) x two light spectra (0 and 20% of FR light in TPFD). Our results showed that the effects of FR light on leaf, stem, and root elongation, leaf number, and leaf expansion were dependent on temperature at lower TPFD. However, the magnitude of the interactive effects between FR light and temperature on plant morphology decreased at higher TPFD. Particularly, at a lower TPFD, FR light stimulated leaf expansion and canopy photon capture only under a cooler temperature of 20°C. However, at a higher TPFD, FR light consistently increased total leaf area across all three temperatures. Plant biomass was more strongly correlated with the total number of photons intercepted by the leaves than with the photosynthetic activities of individual leaves. FR light decreased the contents of chlorophylls, carotenoids, flavonoids, and phenolics, as well as the total antioxidant capacity. In contrast, warmer temperatures and high light intensity increased the values of these parameters. We concluded that the interactive effects between FR light and temperature on plant growth and morphology diminished as total light intensity increased. Additionally, the combination of high light intensity, warm temperature, and FR light resulted in the highest crop yield and antioxidant capacity in lettuce.

## 1 Introduction

Plants can acclimate to various environmental conditions, such as light spectrum and temperature, through specific morphological changes. Under vegetation shade, the ratio of red (R; 600-700 nm) to far-red (FR; 700-800 nm) light decreases because green leaves preferentially absorb R light while transmitting more FR light. An increase in the proportion of FR light, or a decrease in R:FR ratio, can induce adaptive morphological responses including the elongations of hypocotyl, petiole, leaf, and stem; these responses are collectively termed “shade avoidance syndrome” as they enable plants to increase their access to unfiltered light (Smith and Whitelam, 1997; Smith, 2000). Intriguingly, plants exposed to warm temperature often exhibit morphological responses similar to shade-avoiding responses, known as “Thermomorphogenesis” (Casal and Balasubramanian, 2019).

Previous research has found that the morphological changes induced by both FR light and warm temperature are mediated by a common sensor, phytochrome (PHY) photoreceptors (Jung et al., 2016; Legris et al., 2016). Specifically, PHYs undergo interconversion between an active form (P_fr_) and an inactive form (P_r_) when exposed to R and FR light, a process termed “Photoconversion”. In addition to photoconversion, PHYs, particularly photochrome B (PHYB), can convert from the P_fr_ form to the P_r_ form under warm temperatures (“Thermal reversion”) (Jung et al., 2016; Legris et al., 2016). Thus, both light spectrum and temperature can regulate the steady-state of PHYB. When accounting for the dimerization of PHYs, PHYB exists in three states: P_r_-P_r_ (D_0_), P_r_-P_fr_ (D_1_), and P_fr_-P_fr_ (D_2_), where the ratio of active P_fr_ to total PHYs is expressed as D_2_/(D_0_+D_1_+D_2_) because only P_fr_-P_fr_ (D_2_) homodimer is considered biologically active (Brockmann et al., 1987; Klose et al., 2015). Active P_fr_ regulates the activity of its downstream signaling partners, PHYTOCHROME INTERACTING FACTORs (PIFs), which in turn regulate hormonal signaling pathways of gibberellin, auxin, and brassinosteroid, thereby altering plant morphology (Castillon et al., 2007; Franklin, 2008; de Lucas and Prat, 2014).

The dual role of PHYB in sensing both light quality and temperature has been demonstrated in several recent studies, with plant morphology shown to be co-regulated by spectral quality and temperature (Jung et al., 2016; Legris et al., 2016; Romero-Montepaone et al., 2020, 2021; Burko et al., 2022). These studies primarily used Arabidopsis as the model plant and found that FR light-induced hypocotyl elongation of Arabidopsis in response to FR light was enhanced by warm temperature (Romero-Montepaone et al., 2021; Burko et al., 2022). Those studies were conducted under relatively low light intensities (18-100 μmol m^-2^ s^-1^). However, the FR light and temperature interaction may be further dependent on light intensity. Under higher light intensities, photoconversion rates of PHYs accelerate, and the influence of thermal reversion on the PHYB activity is reduced (Sellaro et al., 2019). Additionally, high light intensity can also reduce the rate of thermal reversion by stabilizing the active form of PHYs through nuclear body formation (Ballaré et al., 1991; Chen et al., 2003; Van Buskirk et al., 2014). This might be due to the decrease in the size of the D_1_ (P_r_-P_fr_) heterodimer, which is the main target for thermal reversion, under high light intensity (Klose et al., 2015, 2020; Sellaro et al., 2019). These findings suggest that PHYB activity is predominantly regulated by photoconversion at high light intensities, where thermal reversion has a minimal effect (Sellaro et al., 2019).

Thus, multiple environmental factors, such as FR light, light intensity, and temperature, may interactively affect plant morphology by regulating the steady-state of PHYB. However, previous research mainly focused on quantifying the effects of environmental conditions on the steady-state of PHYB. There has been limited attention given to the subsequent morphological responses mediated by PHYB, with most studies only examining hypocotyl elongation (Jung et al., 2016; Legris et al., 2016; Sellaro et al., 2019; Romero-Montepaone et al., 2020 & 2021; Burko et al., 2022). Furthermore, the interactive effects of environmental factors on plant morphology can be organ-specific (Patel et al., 2013; Jeong et al., 2024). For instance, similar to previous findings in hypocotyl elongation in Arabidopsis, we observed that FR light and warm temperature of 28°C synergistically increased hypocotyl length of lettuce seedlings under a relatively low light intensity of 250 μmol m^-2^ s^-1^ (Jeong et al., 2024). However, lettuce leaf expansion was enhanced by FR light under cooler temperatures (20 or 24°C) but inhibited by FR light under warm temperature of 28°C (Jeong et al., 2024). As leaf expansion is an important determinant of photon capture and biomass accumulation, a decrease in leaf expansion can cause yield reductions (Monteith, 1977; Weraduwage et al., 2015). These findings underscore the need for investigating additional morphological parameters, particularly leaf expansion, when co-optimizing multiple environmental factors to improve crop yield in controlled environment plant production systems. Nonetheless, our current understanding of how light intensity influences the FR light and temperature interactive effect on plant growth and morphology, including stem elongation and leaf expansion, remains limited.

Changes in environmental conditions significantly affect not only morphological traits but also various phytochemicals, including both primary and secondary metabolites (Salam et al., 2023). Extensive research has been conducted to investigate the effects of environmental factors - often focusing on individual factors - on health-promoting compounds and antioxidant capacity in indoor farming systems (Akula and Ravishankar, 2011; Yang et al., 2018; Thoma et al., 2020). For example, high light intensity has been shown to enhance the accumulation of beneficial phytochemicals (Oh et al., 2009; Tattini et al., 2014; Dou et al., 2018; Pérez-López et al., 2018). Similarly, warm temperature can increase the levels of chlorophylls, carotenoids, flavonoids, phenolics, and antioxidant capacity (Lefsrud et al., 2005; Oh et al., 2009; Shamloo et al., 2017). In contrast, FR light tends to decrease chlorophylls (Lefsrud et al., 2008; Li and Kubota, 2009; Kong and Nemali, 2021), carotenoids (Lefsrud et al., 2008; Kong and Nemali, 2021; Meng et al., 2024), flavonoids (Oh et al., 2021), phenolics (Li and Kubota, 2009; Bantis et al., 2016; Oh et al., 2021), and antioxidant capacity (Oh et al., 2021). Given the differential effects of light intensity, temperature, and FR light on phytochemical accumulation and antioxidant capacity, comprehensive studies exploring the interactions among these environmental factors are needed to develop effective strategies for improving plant nutritional quality.

Through this integrative experiment with three different environmental factors (i.e., light intensity, temperature, and FR light), our objectives were 1) to investigate how light intensity affects the interactive effects between FR light and warm temperature on lettuce growth and morphology, 2) to determine how various physiological and biochemical parameters, including photosynthesis, pigmentation, secondary metabolites, and antioxidant capacity, respond to different light intensities, temperatures, and FR light, and 3) to identify potentially optimal combinations of these three environmental factors that maximize both crop yield and nutritional quality, particularly antioxidant capacity.

## 2 Materials and methods

### 2.1 Plant materials

Three seeds of lettuce (*Lactuca sativa* L.) ‘Rex’ (Johnny’s Selected Seeds, Winslow, ME, USA) were sown in 0.45 L plastic pots (8.8 cm x 8.8 cm x 8.9 cm; length x width x height) filled with a soilless substrate (BM6; peat-moss and perlite; Berger, Saint-Modeste, QC, Canada) in a glass-covered greenhouse. Seedlings were moved into growth chambers six days after sowing. The seedlings were thinned to one plant per pot based on uniformity. Plants were manually irrigated with a nutrient solution containing 150 mg L^-1^ N and other essential mineral nutrients made with water-soluble fertilizer (21N-2.2P-16.6K; Peters 21-5-20; The Scotts Company, Marysville, OH, USA) throughout the experiment.

### 2.2 Light and temperature treatments

**Figure 1:**
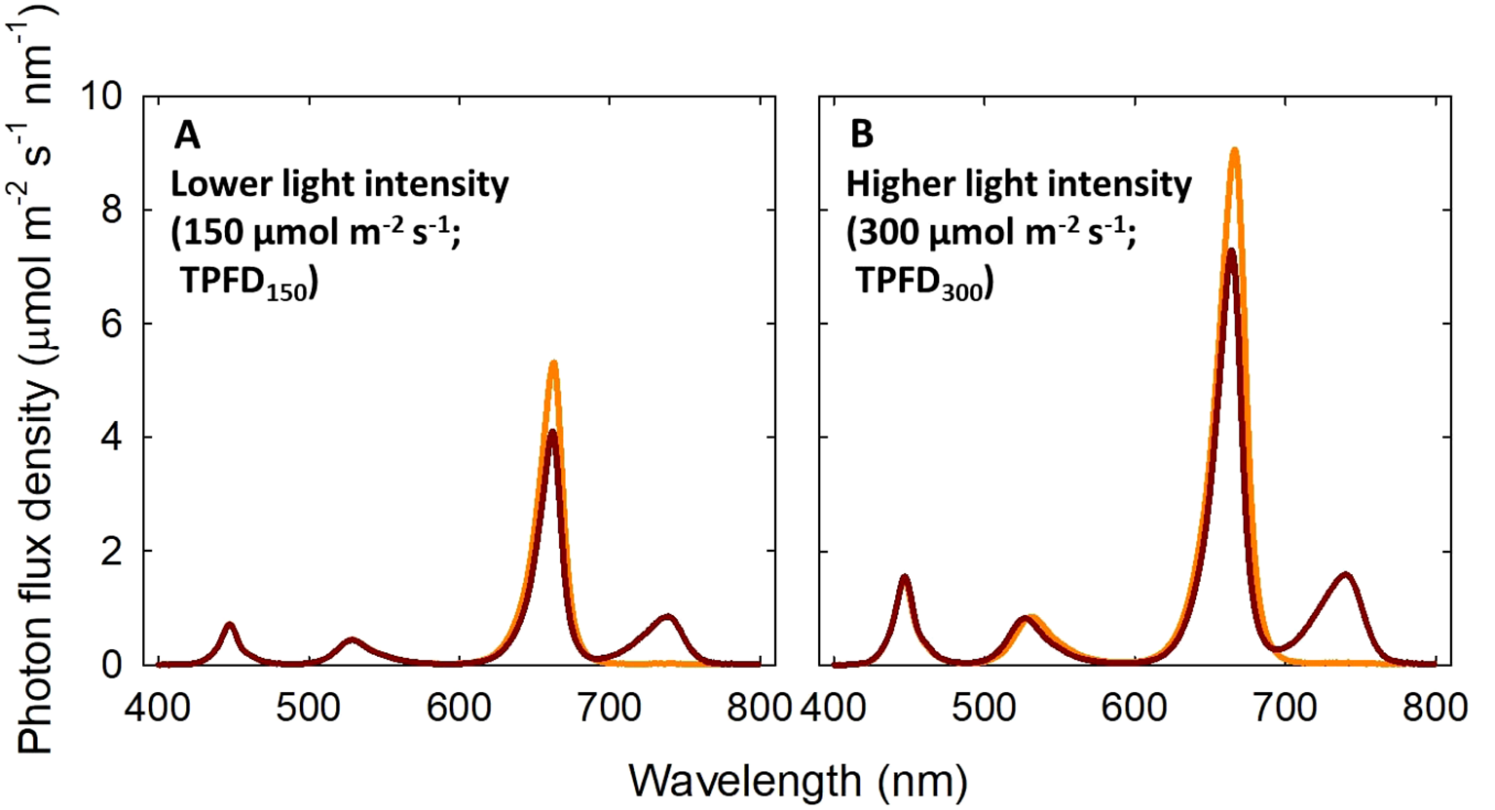
Spectral distributions of four light treatments consisted of blue (B; 400-500 nm), green (G; 500-600 nm), red (R; 600-700 nm), and far-red (FR; 700-800 nm) photons delivered by light-emitting diodes. Two total photon flux densities (TPFD; 400 to 800 nm) were used: μmol m^-2^ s^-1^ **(A)** and 300 μmol m^-2^ s^-1^ **(B)**. Two light spectral treatments were denoted based on the percentage of FR photons in TPFD: 0%FR (B_15_G_15_R_120_) and 20%FR (B_15_G_15_R_90_FR_30_) under TPFD of 150 μmol m^-2^ s^-1^ and 0%FR (B_30_G_30_R_240_) and 20%FR (B_30_G_30_R_180_FR_60_) under TPFD of 300 μmol m^-2^ s^-1^. The subscript after each waveband indicates its photon flux density in μmol m^-2^ s^-1^.

In this experiment, three walk-in growth chambers were used to establish three temperature setpoints: 20°C, 24°C, and 28°C (actual temperature was 21.2 ± 1.4°C, 24.4 ± 1.2°C, and 28.9 ± 1.0°C, respectively). This temperature range supports normal growth and development of lettuce ‘Rex’ without causing chilling or heat stress symptoms (Jeong et al., 2024). In each walk-in growth chamber, four sections (l x w x h; 60 x 60 x 70 cm) were created using growth-racks and reflective cardboards to accommodate four light treatments: two total photon flux densities (TPFDs; 400-800 nm) (150 or 300 μmol m^-2^ s^-1^) x two FR light levels (0 or 20% of FR light in TPFD). The corresponding light treatments were as follows: 0% FR (B_15_G_15_R_120_) and 20% FR (B_15_G_15_R_90_FR_30_) of FR light at a lower TPFD of 150 μmol m^-2^ s^-1^ (TPFD_150_) and 0% FR (B_30_G_30_R_240_) and 20% FR (B_30_G_30_R_180_FR_60_) at higher TPFD of 300 μmol m^-2^ s^-1^ (TPFD_300_). B stands for blue light (400-500 nm) and G stands for green light (500-600 nm) (**Figure 1** and **Supplementary Table 1**). The subscript after each waveband indicates its photon flux density in μmol m^-2^ s^-1^. The spectral treatments were created using an LED research lighting system (PHYTOFY^®^ RL, Osram, Munich, Germany) and the intensity of each light spectrum was adjusted using PHYTOFY^®^ RL software (version 1.0.8). The peak wavelengths were 450 nm for B, 521 nm for G, 660 nm for R, and 730 nm for FR LEDs. All treatments had a 24-h photoperiod. The daily light integral (DLI) of the treatments with a TPFD of 150 and 300 μmol m^-2^ s^-1^ was 13 and 26 mol m^-2^ d^-1^, respectively. The two light intensity levels were selected based on the recommended DLI range of 10 to 20 mol m^-2^ d^-1^ for indoor lettuce cultivation (Yan et al., 2019; Kelly et al., 2020; Matysiak et al., 2022). The FR fraction [FR/(R+FR); Kusuma and Bugbee, 2021] in the treatments with 20% FR light was 0.25.

Temperature in each growth chamber section was measured every 30 seconds and recorded every 20 minutes using type-E thermocouples connected to a data logger (CR1000; Campbell Scientific, Logan, UT, USA). Within each treatment area, photon flux density (30 cm distance from LEDs to the top of plant) was measured at fourteen locations (see **Supplementary Table 1** for the standard deviation of the light intensity under each treatment) with a spectroradiometer (PS100; Apogee Instruments, Logan, UT, USA). To ensure consistent light intensity at the top of the plants, a constant distance between the LEDs and plant canopy was maintained by periodically lowering the shelves. To minimize any effects caused by spatial variation in light intensity, plants within each treatment were randomly rotated daily.

### 2.3 Data collection and analysis

#### 2.3.1 Morphological and growth parameters

Plants were harvested 20 days after treatment (DAT). Various vegetative parameters were measured at harvest to assess plant growth and morphology. Total leaf number, stem length, and leaf length and width of the most recently mature leaf were recorded. Leaf length:width ratio was calculated. Stem length was obtained after detaching all the leaves. Total leaf area was measured using a leaf area meter (LI-3100C; LI-COR, Lincoln, NE). Total leaf area and stem length were divided by the growth period to calculate the average leaf expansion rate and the average stem elongation rate, respectively. For root morphological analysis, lettuce roots were submerged in water and gently washed. Then, the roots were scanned using Epson Perfection V850 Pro (Seiko Epson Corporation; Suwa, Japan). Total root length and average root diameter were measured using WinRHIZO root analysis software (Regent Instruments, Quebec, QC, Canada). Leaf, stem, and root fresh weights (FWs) were recorded. Subsequently, the dry weight (DW) of each plant organ was determined after oven-dried at 80°C for seven days. Total DW was calculated as the sum of leaf, stem, and root DWs. Total number of photons intercepted by each plant was estimated using top-down photos of lettuce. The top-down photos were taken every five days (0, 5, 10, 15, and 20 DAT) using digital camera placed 150 cm from the plants. Then, the projected leaf area was measured with ImageJ software (National Institutes of Health). Total intercepted photons were calculated with the projected leaf area following the method described in Legendre and van Iersel (2021).

#### 2.3.2 Photosynthetic parameters

To quantify leaf photosynthetic efficiency under the treatment conditions, chlorophyll fluorescence and CO_2_ exchange rates were measured on the most recently fully expanded leaves one to three days prior to harvest.

Leaf chlorophyll fluorescence was measured using a chlorophyll fluorometer (OS5p; Opti-Science, Inc., Hudson, NH, USA). Leaves were dark-adapted for 30 minutes using dark adaptation clips to determine the minimum fluorescence (*F_o_*). A saturating light pulse was then applied to measure the maximum fluorescence (*F_m_*). The maximum quantum efficiency of PSII photochemistry was calculated as *F_v_*/*F_m_*, where *F_v_* represents the variable fluorescence (*F_v_ = F_m_* − *F_o_*). To evaluate photochemical efficiency under treatment light conditions, maximum fluorescence (*F_m_’*) and steady-state fluorescence levels (*F’*) were measured on the leaves adapted to the light condition of each treatment. The quantum yield of PS II (*Φ_PSII_*) was calculated using the formula (*F_m_’* - *F’*)/*F_m_’* (Baker, 2008).

Additionally, net CO_2_ assimilation rate (*P_net_*) and dark respiration rate (*R_d_*) were measured using a portable gas exchange analyzer (CIRAS-3; PP Systems, Amesbury, MA, USA) equipped with a PLC3 leaf cuvette, which features a clear top chamber (25 mm x 18 mm). The measurement was made under treatment light conditions. The CO_2_ concentration in the cuvette was maintained at 390 μmol mol^-1^, and the air temperature in the cuvette was set to match the treatment temperature (20, 24, or 28°C).

#### 2.3.3 Pigments, secondary metabolites, and antioxidant capacity

To quantify the levels of pigments, secondary metabolites, and antioxidant capacity, the most recently fully expanded leaves were sampled at midday one day before harvest (19 DAT) in 2^nd^ replicate. The samples were immediately immersed into liquid nitrogen, homogenized with mortar and pestle, and then stored in a −80°C freezer (IU1786A, Thermo Fisher Scientific, Waltham, MA, USA) until phytochemical analysis.

To determine the chlorophyll and carotenoid contents, 50 mg of fresh samples were incubated in 1.5 ml of pure methanol for 24 hours. Then, the samples were centrifuged at 10,000 g for 10 min to separate the supernatant. The absorbance of the supernatant was measured at 470 nm, 652 nm, and 665 nm using a spectrophotometer (Genesys 10S UV-Vis; Thermo Fisher Scientific, Waltham, MA, USA). The concentration of chlorophylls and carotenoids were then calculated according to the protocol outlined by Wellburn (1994).

The determination of the levels of secondary metabolites and antioxidant capacity were conducted following the method described in Dou et al. (2019). For this analysis, 100 mg of fresh samples were extracted using 0.75 ml of 1% acidified methanol at 4°C in darkness. After a 12-h extraction, the samples were centrifuged at 10,000 g for 10 min to collect the supernatant for further analysis. For the quantification of phenolic content, a modified Folin-Ciocalteu reagent method was employed. In this method, 100 μl of the extract was mixed with 150 μl of distilled water and 750 μl of a 1/10 dilution of the Folin-Ciocalteu reagent. After a 6-min reaction period, 600 μl of 7.5% Na_2_CO_3_ solution was added to the mixture. The mixture was then incubated at 45°C in a water bath for 10 minutes, and the absorbance was measured at 725 nm using the microplate reader (ELx800, BioTek, Winooksi, VT, USA). The phenolic content was expressed as milligrams of gallic acid equivalent per gram of FW. Flavonoid content was determined by mixing 20 μl of the extract with 85 μl of distilled water and 5 μl of 5% NaNO_2_. After a 6-min reaction, 10 μl of 10% AlCl_3_·6H_2_O was added. Five minutes later, 35 μl of 1 M NaOH and 20 μl of distilled water were added to the mixture. The absorbance was measured at 520 nm using the microplate reader (ELx800), and the flavonoid content was expressed as milligrams of (+)-catechin hydrate equivalent per gram of FW. The antioxidant capacity was assessed using the ABTS method as described by Arnao et al. (2001). The 150 μl of the extract was mixed with 2.85 ml of the colored ABTS+ solution. After a 10-minute reaction at room temperature, the absorbance was measured at 734 nm using the microplate reader (ELx800). The antioxidant capacity results were expressed as milligrams of Trolox equivalent antioxidant capacity per gram of FW.

#### 2.3.4 Estimation of PPE based on a simplified three-state model

In our study, the proportion of active P_fr_ in total PHYB [D_2/_(D_0_ + D_1_ + D_2_)] was calculated using a simplified three-state model (Klose et al., 2015; Sellaro et al., 2019):


(1) $$\begin{array}{c} {\text{D}}_{2}/({\text{D}}_{0}+{\text{D}}_{1}+{\text{D}}_{2})\\ =\frac{2{\text{k}}_{1}^{2}}{2{\text{K}}_{1}^{2}+\;2{\text{k}}_{1}\left(2{\text{k}}_{2}+\;2{\text{k}}_{\text{r}2}\right)+\left({\text{k}}_{2}+{\text{ k}}_{\text{r}1}\right)\left(2{\text{k}}_{2}+\;2{\text{k}}_{\text{r}2}\right)} \end{array}$$

Where k_1_ and k_2_ are rate constants for phytochrome photoconversion, calculated from the incident light spectral photon flux (**Figure 1**) and the phytochrome photoconversion coefficients:


(2) $${\text{k}}_{1}=\sum_{\text{λ}=300\text{nm}}^{\text{λ}=800\text{nm}}{\text{I}}_{\text{λ}}{\text{σ}}_{\text{R},\text{λ}}$$

(3) $${\text{k}}_{2}=\sum_{\text{λ}=300\text{nm}}^{\text{λ}=800\text{nm}}{\text{I}}_{\text{λ}}{\text{σ}}_{\text{FR},\text{λ}}$$

I_λ_ is the incident photon flux density at wavelength λ. σ_R,λ_ is the photoconversion coefficient for the conversion of P_r_ to P_fr_ and σ_FR,λ_ is the photoconversion coefficient for the conversion of P_fr_ to P_r_ at wavelength λ (Sager et al., 1988).

Additionally, in Equation 1, k_r1_ is the thermal reversion rate of D_1_ to D_0_ and k_r2_ is the thermal reversion rate of D_2_ to D_1._ In our study, we only considered the effect of photoconversions in the estimation of PPE by setting k_r1_ = k_r2_ = 0 in Equation 1 (Sellaro et al., 2019). Therefore, the calculated value represents the proportion of D_2_ in total PHYB at photoequilibrium, assuming that the effect of thermal reversion is negligible. In our study, the estimated PPE was 0.79 under 0% FR light and 0.67 under 20% FR light at both light intensity levels.

### 2.4 Experimental design and statistical analysis

This experiment was replicated two times. In each replicate, four plants (subsamples) per treatment were used in each of the twelve treatments [two light intensities (150 or 300 μmol m^-2^ s^-1^) x three temperatures (20, 24, or 28°C) x two light spectra (0 or 20% FR)]. A split-plot block design was employed, where the main-plot factor was temperature and the sub-plot factor was light spectral quality. The chamber temperature set points and the locations of spectral treatments were randomized in each replicate. Three- and two-way analysis of variance (ANOVA) procedure was utilized to analyze the data using the Statistical Analysis System version 9.4 (SAS Inst., Inc., Cary, NC, USA). Subsamples were averaged before statistical analysis. Significant difference among the treatments was determined using Duncan’s multiple range test at *p*< 0.05. SigmaPlot software version 12.5 (Systat Software, Inc., Chicago, IL, USA) was used for regression analyses.

## 3 Results

### 3.1 Plant morphology

**Figure 2:**
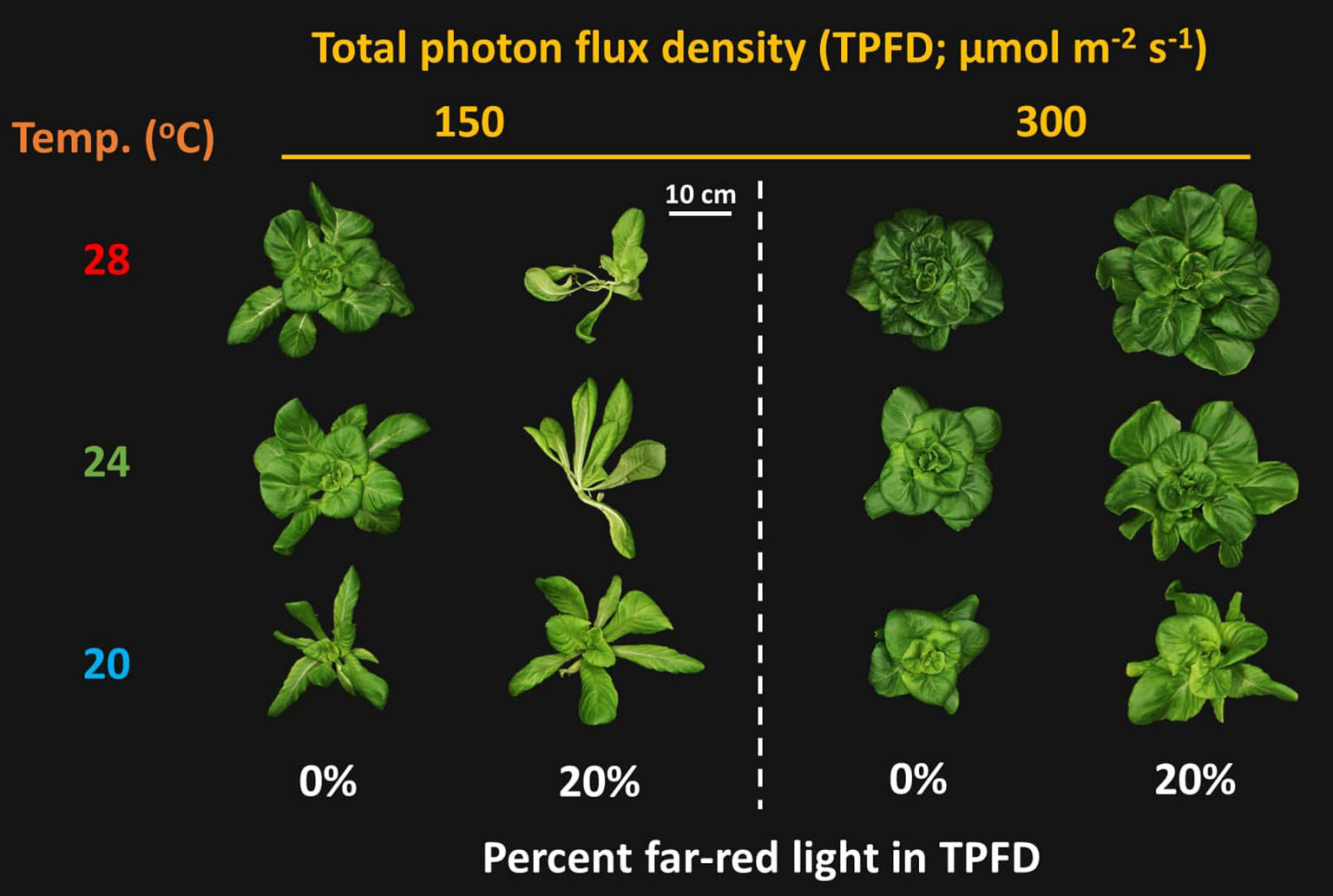
Representative lettuce shoots grown under two light intensities x three temperatures x two light spectra. The two light intensities were total photon flux densities (TPFDs; 400 to 800 nm) of 150 and 300 μmol m^-2^ s^-1^. The three temperatures were 20, 24, and 28°C. The two light spectra are denoted based on the percentage of far-red photons (FR; 700-800 nm) in TPFD, i.e., 0 and 20% FR light.

**Figure 3:**
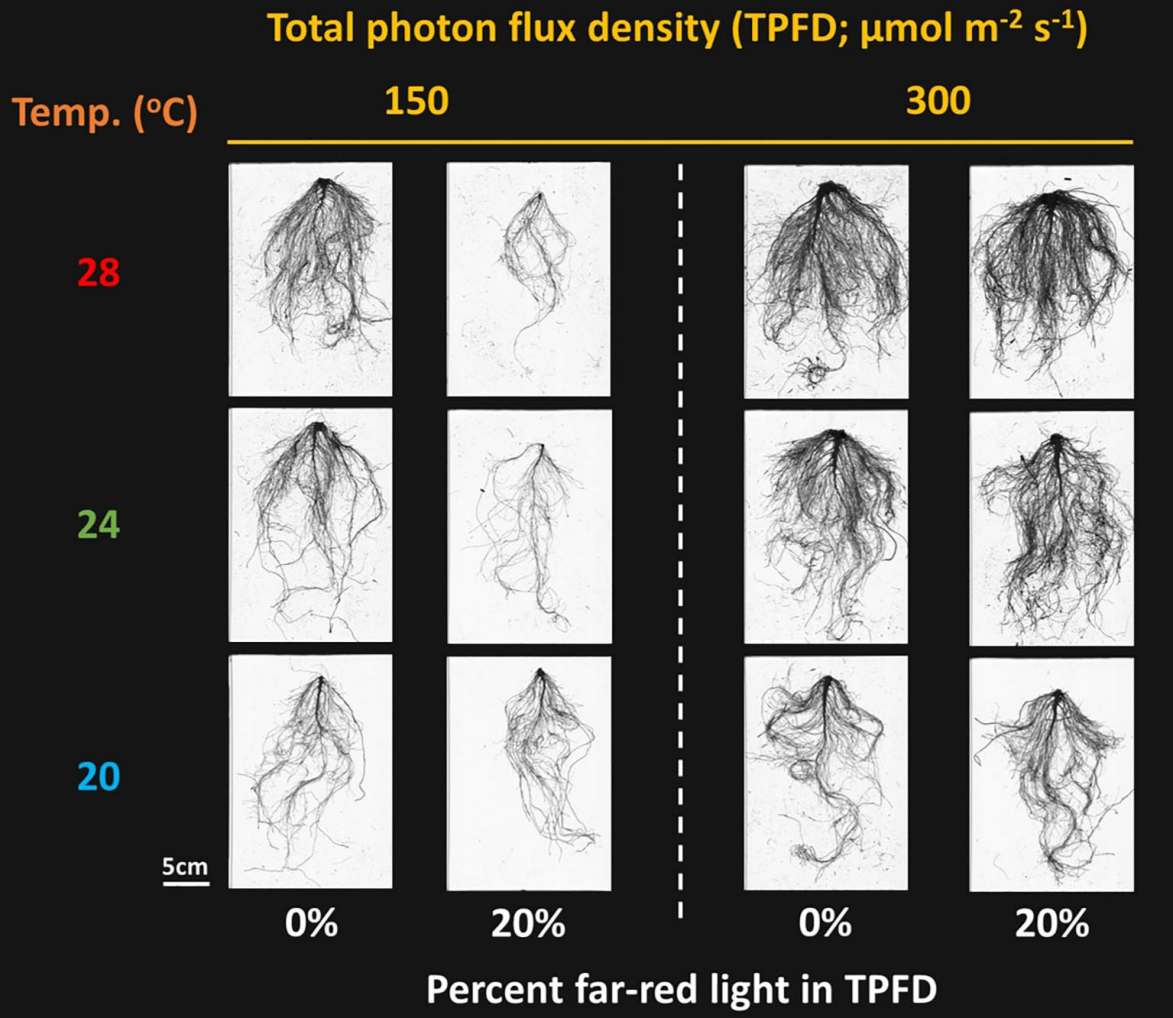
Representative lettuce roots grown under two light intensities x three temperatures x two light spectra. The two light intensities were total photon flux densities (TPFDs; 400 to 800 nm) of 150 and 300 μmol m^-2^ s^-1^. The three temperatures were 20, 24, and 28°C. The two light spectra are denoted based on the percentage of far-red photons (FR; 700-800 nm) in TPFD, i.e., 0 and 20% FR light.

**Figure 4:**
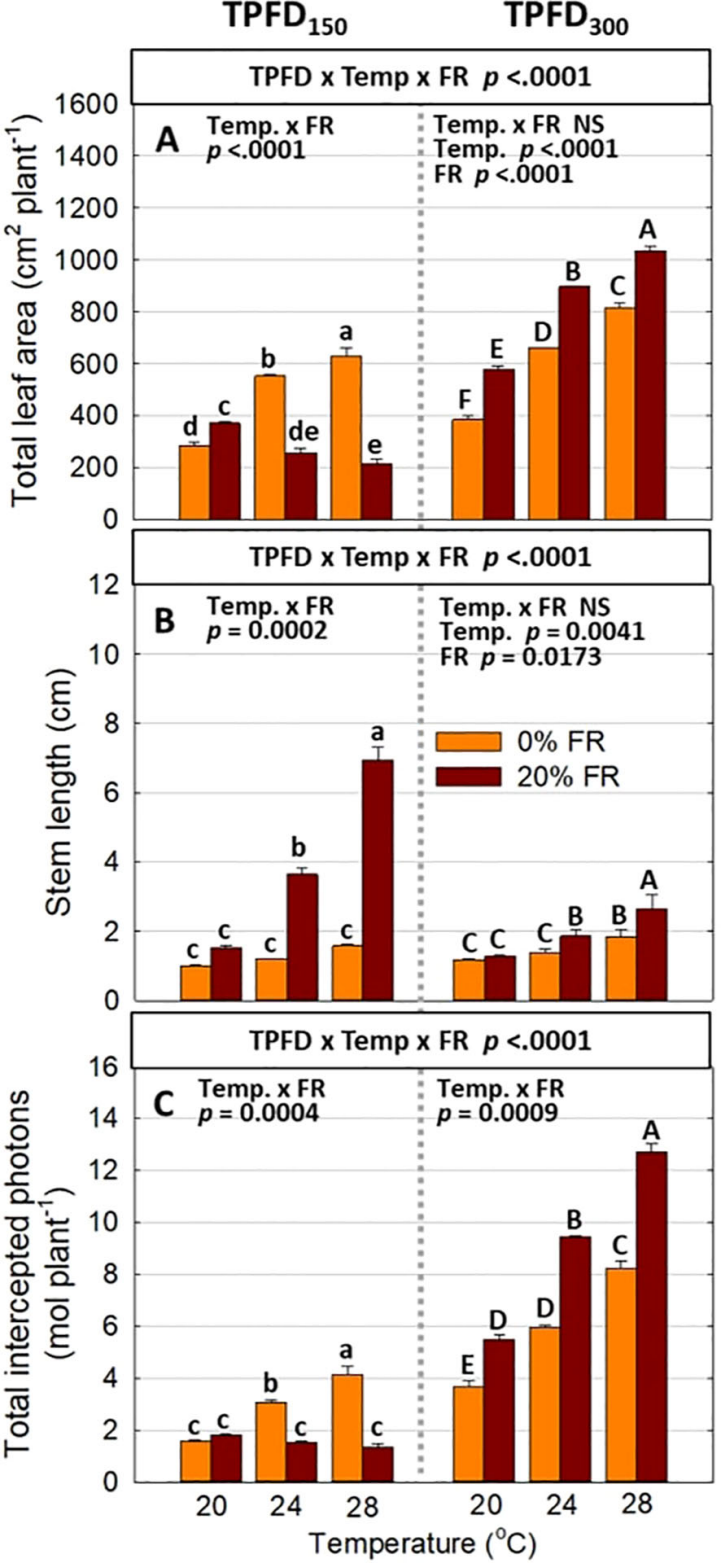
The interactive effect between light spectra and temperature under two light intensities [150 and 300 μmol m^-2^ s^-1^ in total photon flux density (TPFD; 400-800 nm)] on total leaf area **(A)**, stem length **(B)**, and total intercepted photons **(C)**. The two light spectra are denoted based on the percentage of far-red photons (FR; 700-800 nm) in TPFD, i.e., 0 and 20% FR light. The three temperatures were 20, 24, and 28°C. Different letters following the mean ± SE [n = 2; subsamples (4 plants per treatment per replicate study) were averaged before statistical analysis] indicate significant difference among the six treatments (three temperatures x two FR levels) at each light intensity at *p*< 0.05. NS stands for non-significance.

**Figure 5:**
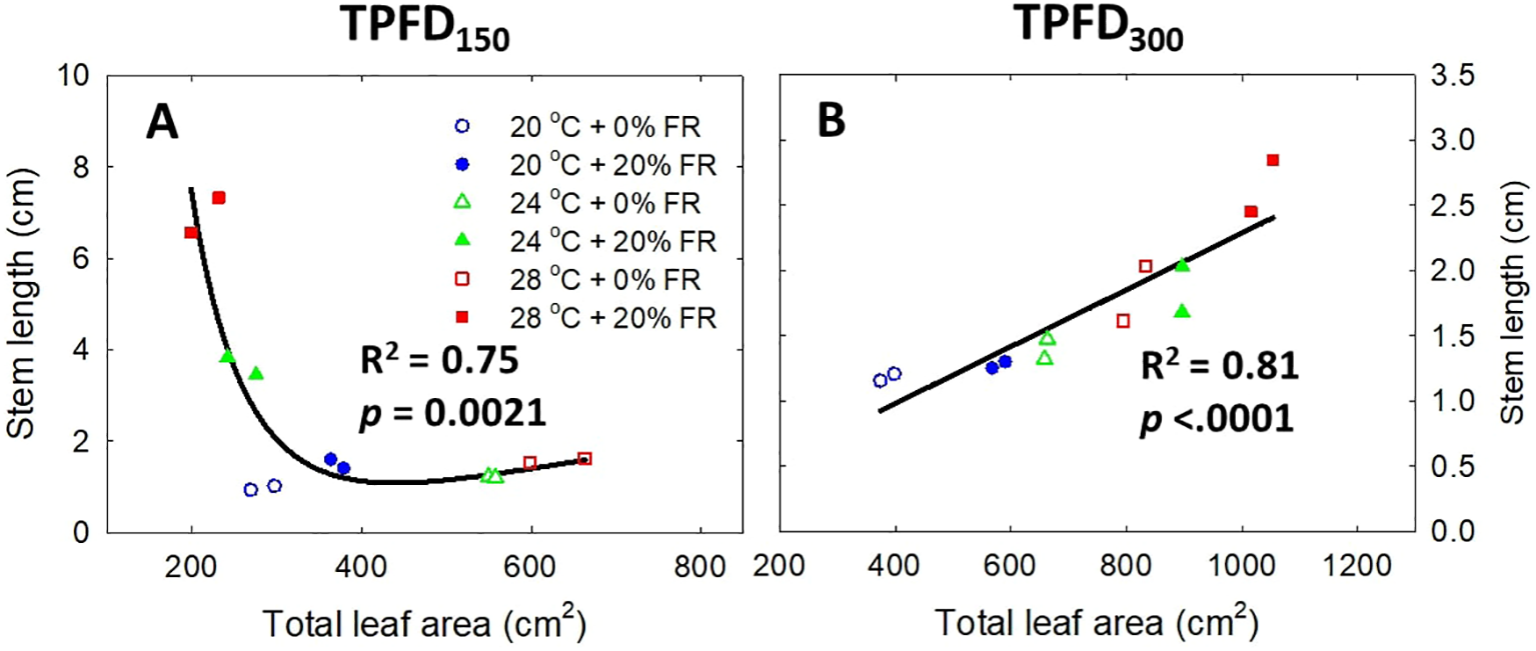
Relationship between total leaf area and stem length under the interactive effect between light spectra and temperature under two light intensities of 150 μmol m^-2^ s^-1^ **(A)** and 300 μmol m^-2^ s^-1^ **(B)** in total photon flux density (TPFD; 400-800 nm). The two light spectra are denoted based on the percentage of far-red photons (FR; 700-800 nm) in TPFD, i.e., 0 and 20% FR light. The three temperatures were 20, 24, and 28°C.

Overall, we found that FR light and temperature interactively regulated lettuce growth and morphology at a lower TPFD of 150 μmol m^-2^ s^-1^; however, increasing TPFD from 150 to 300 μmol m^-s2^ s^-1^ caused the interaction between FR light and temperature to diminish (**Figures 2**–**5** and **Supplementary Figures 1**, **2**).

#### 3.1.1 Shoot morphology and total intercepted photons

Significant three-way interactive effects among TPFD, temperature, and FR light were observed on shoot morphological parameters (**Figure 4** and **Supplementary Figures 1C, D**). In particular, light intensity influenced how FR light and temperature interacted to regulate plant morphology. Two-way ANOVA revealed a significant interaction between FR light and temperature on leaf and stem elongation and leaf expansion at low light intensity (TPFD_150_), but not at high light intensity (TPFD_300_) (**Figure 4** and **Supplementary Figures 1C, D**). Specifically, at TPFD_150_, FR light stimulated leaf expansion at cooler temperature (20°C) but decreased total leaf area and total leaf number under warmer temperatures (24°C and 28°C) (**Figure 4A** and **Supplementary Figure 1D**). FR light increased stem elongation at all three temperatures at TPFD_150_. The stimulative effect of FR light on stem elongation was much more pronounced at warmer temperatures than at cool temperature of 20°C (**Figure 4B**). However, at TPFD_300_, the magnitude of the interactive effects between FR light and temperature on total leaf area, stem length, leaf length:width ratio, and total leaf number diminished, and the effects of FR light on these morphological traits were similar across all three temperatures (**Figures 4A, B** and **Supplementary Figures 1C, D**). For example, at TPFD_150_, substituting 20% FR light for R light resulted in a 30% increase in total leaf area at 20°C but a 53% decrease at 24°C and a 66% decrease at 28°C (**Figure 4A**). At TPFD_300_, FR light significantly increased total leaf area at all temperatures, by 50% increase at 20°C, 36% increase at 24°C, and 27% increase at 28°C (**Figure 4A**). Stem elongation and total leaf area showed a negative correlation at TPFD_150_, but a positive correlation at TPFD_300_ (**Figure 5**). Total intercepted photons showed similar responses to light intensity, temperature, and FR light as total leaf area (**Figure 4C**).

#### 3.1.2 Root morphology

Significant three-way interactive effects among TPFD, temperature, and FR light were observed on total root length and average root diameter (**Supplementary Figure 2**). Similar to the plant shoot morphological parameters, FR light and temperature interactively regulated root morphology at TPFD_150_; however, this interactive effect disappeared at TPFD_300_ (**Figure 3** and **Supplementary Figure 2**). For example, at TPFD_150_, the substitution of 20% FR light for R light did not affect total root length at 20°C but significantly decreased these parameters at warmer temperatures of 24°C and 28°C (**Supplementary Figure 2A**). Applying 20% FR light at TPFD_150_ also caused a significant decrease in the average root diameter at warmer temperatures of 24°C and 28°C, but not at 20°C (**Supplementary Figure 2B**). However, at TPFD_300_, FR light had no effect on total root length across all three temperatures, and the average root diameter increased under warm temperature regardless of FR light (**Supplementary Figure 2**).

### 3.2 Plant biomass

**Figure 6:**
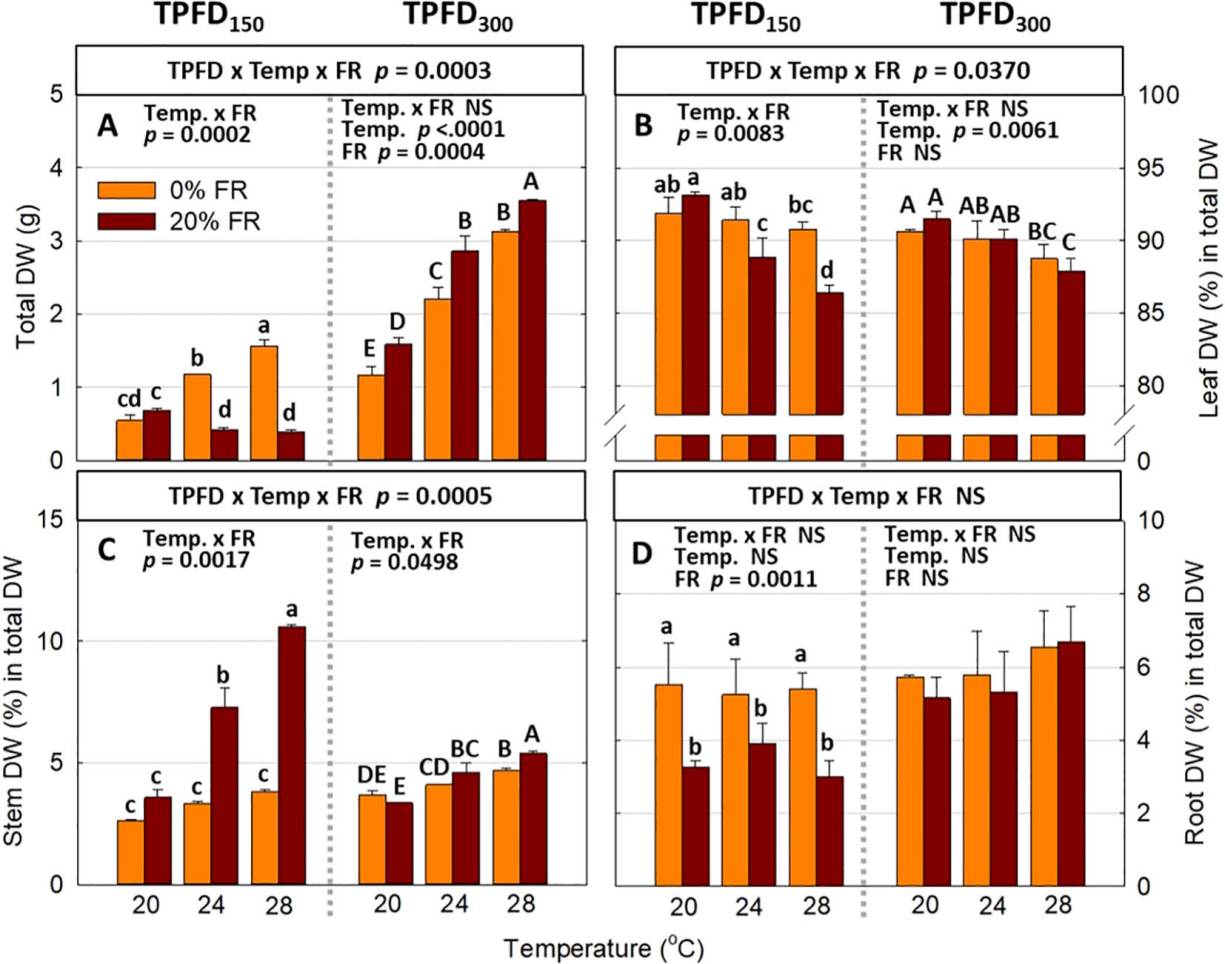
The interactive effect between light spectra and temperature under two light intensities [150 and 300 μmol m^-2^ s^-1^ in total photon flux density (TPFD; 400-800 nm)] on total dry weight (DW) **(A)**, leaf DW (%) in total DW **(B)**, stem DW (%) in total DW **(C)**, and root DW (%) in total DW **(D)**. The two light spectra are denoted based on the percentage of far-red photons (FR; 700-800 nm) in TPFD, i.e., 0 and 20% FR light. The three temperatures were 20, 24, and 28°C. Different letters following the mean ± SE [n = 2; subsamples (4 plants per treatment per replicate study) were averaged before statistical analysis] indicate significant difference among the six treatments (three temperatures x two FR levels) at each light intensity at *p*< 0.05. NS stands for non-significance.

Significant three-way interactions among light intensity, FR light, and temperature were also observed in plant biomass parameters, including the FW and DW of leaf, stem, root, and total plant mass (**Figure 6A** and **Supplementary Figure 3**). Consistent with morphological parameters, FR light and temperature interactively regulated plant biomass accumulation and partitioning at low light intensity (TPFD_150_), but the magnitude of the interactive effects diminished at high light intensity (TPFD_300_). Specifically, the response of total plant DW to FR light followed a similar pattern as total leaf area (**Figure 6A**). At TPFD_150_, increasing the FR light from 0 to 20% had no significant effect on total leaf area at 20°C but caused a 63% decrease at 24°C and a 75% decrease at 28°C (**Figure 6A**). However, at TPFD_300_, substituting FR light for R light significantly increased shoot DW under all three temperatures, by 37% at 20°C, 29% at 24°C, and 14% at 28°C (**Figure 6A**). Regarding biomass partitioning, at TPFD_150_, FR light did not affect percent leaf DW at 20°C, but significantly decreased percent leaf DW at warmer temperatures of 24°C and 28°C. In contrast, at TPFD_300_, percent leaf DW was not affected by FR light regardless of temperature conditions (**Figure 6B**). In contrast to percent leaf DW responses, at TPFD_150_, the stimulative effect of FR light on percent stem DW was greater at warm temperatures, with no significant increase at 20s°C, a 118% increase at 24°C, and a 179% increase at 28°C (**Figure 6C**). However, at TPFD_300_, the magnitude of the interactive effects between FR light and temperature on percent stem DW diminished, and FR light was less effective on percent stem DW than those at a lower TPFD (**Figure 6C**). Percent root DW showed no significant interaction among TPFD, temperature, and FR light, but FR light significantly decreased percent root at lower TPFD (**Figure 6D**).

### 3.3 Photosynthesis in single-leaf level

**Figure 7:**
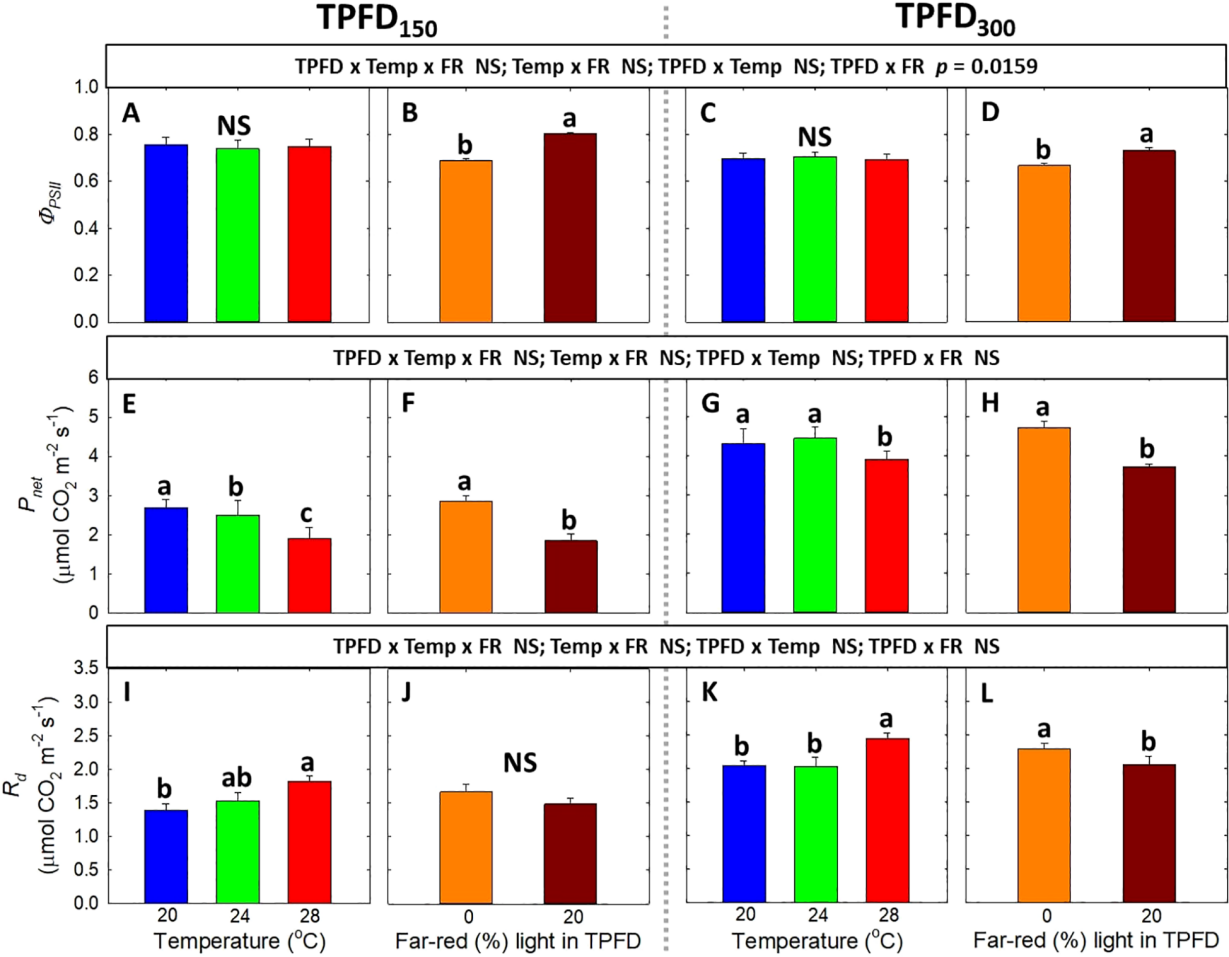
The interactive effect between light spectra and temperature under two light intensities [150 and 300 μmol m^-2^ s^-1^ in total photon flux density (TPFD; 400-800 nm)] on quantum yield of photosystem II (*Φ_PSII_*) **(A–D)**, net CO_2_ assimilation rate (*P_net_*) **(E–H)**, and dark respiration rate (*R_d_*) **(I–L)**. The two light spectra are denoted based on the percentage of far-red photons (FR; 700-800 nm) in TPFD, i.e., 0 and 20% FR light. The three temperatures were 20, 24, and 28°C. Different letters following the mean ± SE [n = 2; subsamples (4 plants per treatment per replicate study) were averaged before statistical analysis] indicate significance at *p*< 0.05. NS stands for non-significance.

Neither three-way interaction among light intensity, temperature, and FR light, nor two-way interaction between FR light and temperature, was observed in any of the photosynthetic parameters, including *Φ_PSII_*, *P_net_*, and *R_d_* (**Figure 7**). At both TPFD levels, FR light significantly increased *Φ_PSII_*, whereas temperature had no effect on *Φ_PSII_* (**Figures 7A–D**). Both warm temperature (28°C) and FR light caused *P_net_* to decrease under each TPFD level (**Figures 7E–H**). *R_d_* significantly increased with increasing temperature from 20°C to 28°C but decreased under 20% FR light (**Figures 7I–L**).

**Figure 8:**
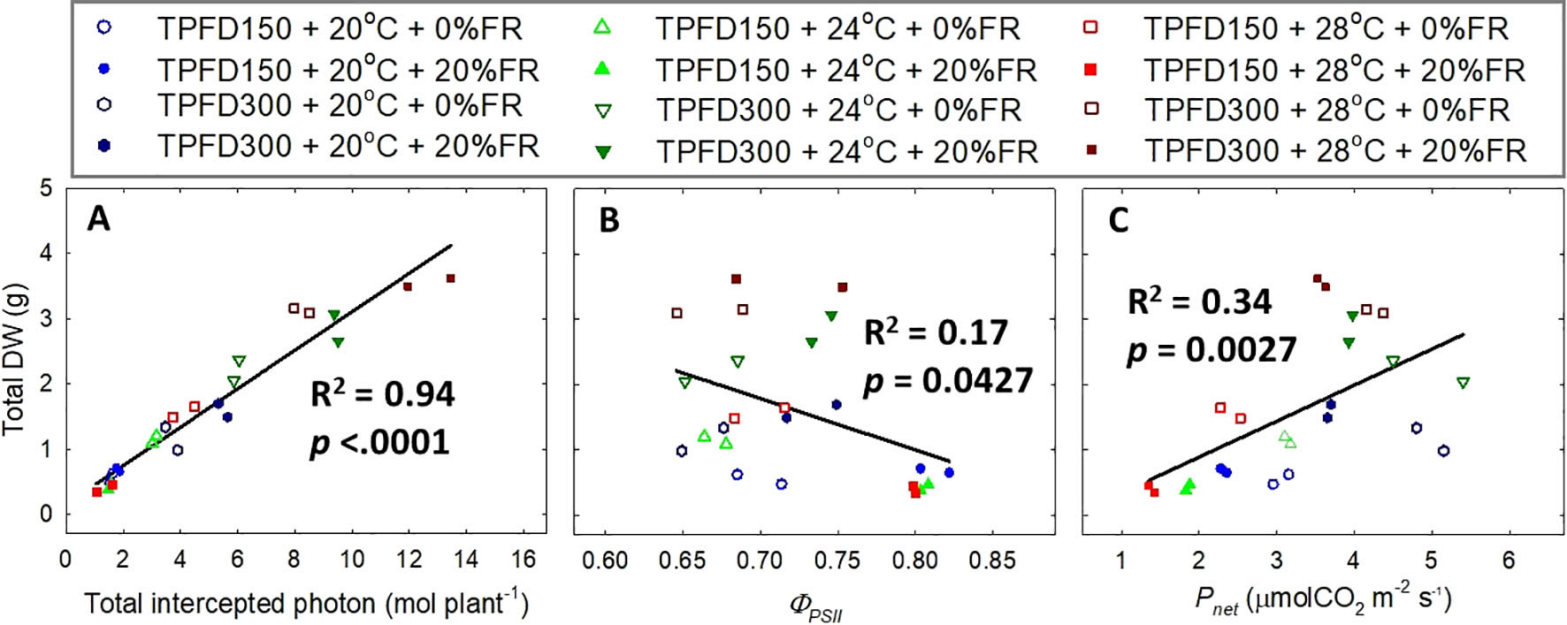
Relationship of total intercepted photons **(A)**, quantum yield of photosystem II (*Φ_PSII_*) **(B)**, and net CO_2_ assimilation rate (*P_net_*) **(C)** with total dry weight (DW) under the interactive effect between light spectra and temperature under two light intensities [150 and 300 μmol m^-2^ s^-1^ in total photon flux density (TPFD; 400-800 nm)]. The two light spectra are denoted based on the percentage of far-red photons (FR; 700-800 nm) in TPFD, i.e., 0 and 20% FR light. The three temperatures were 20, 24, and 28°C.

Total DW was positively correlated with total intercepted photons and *P_net_*, but it was negatively correlated with *Φ_PSII_* (**Figure 8**). Total intercepted photons showed much higher R^2^ value of 0.94, compared to those for *Φ_PSII_* (R^2^ = 0.17), and *P_net_* (R^2^ = 0.34).

### 3.4 Chlorophyll contents

Similar to the photosynthetic parameters, there was no significant three-way interaction among the three environmental factors, nor was there any significant two-way interaction between FR light and temperature in the chlorophyll contents and chlorophyll a:b ratio (**Supplementary Figure 4**). At both TPFD levels, chlorophyll a content significantly increased with increasing temperature from 20°C to 28°C, but decreased at 20% FR light (**Supplementary Figures 4A–D**). Warm temperature of 28°C also significantly increased chlorophyll b content, although FR light did not affect chlorophyll b contents at either TPFD level (**Supplementary Figures 4E–H**). Chlorophyll a:b ratio significantly decreased by warm temperature (28°C) and FR light at both TPFDs (**Supplementary Figures 4I–L**).

### 3.5 Secondary metabolites and antioxidant capacity

**Figure 9:**
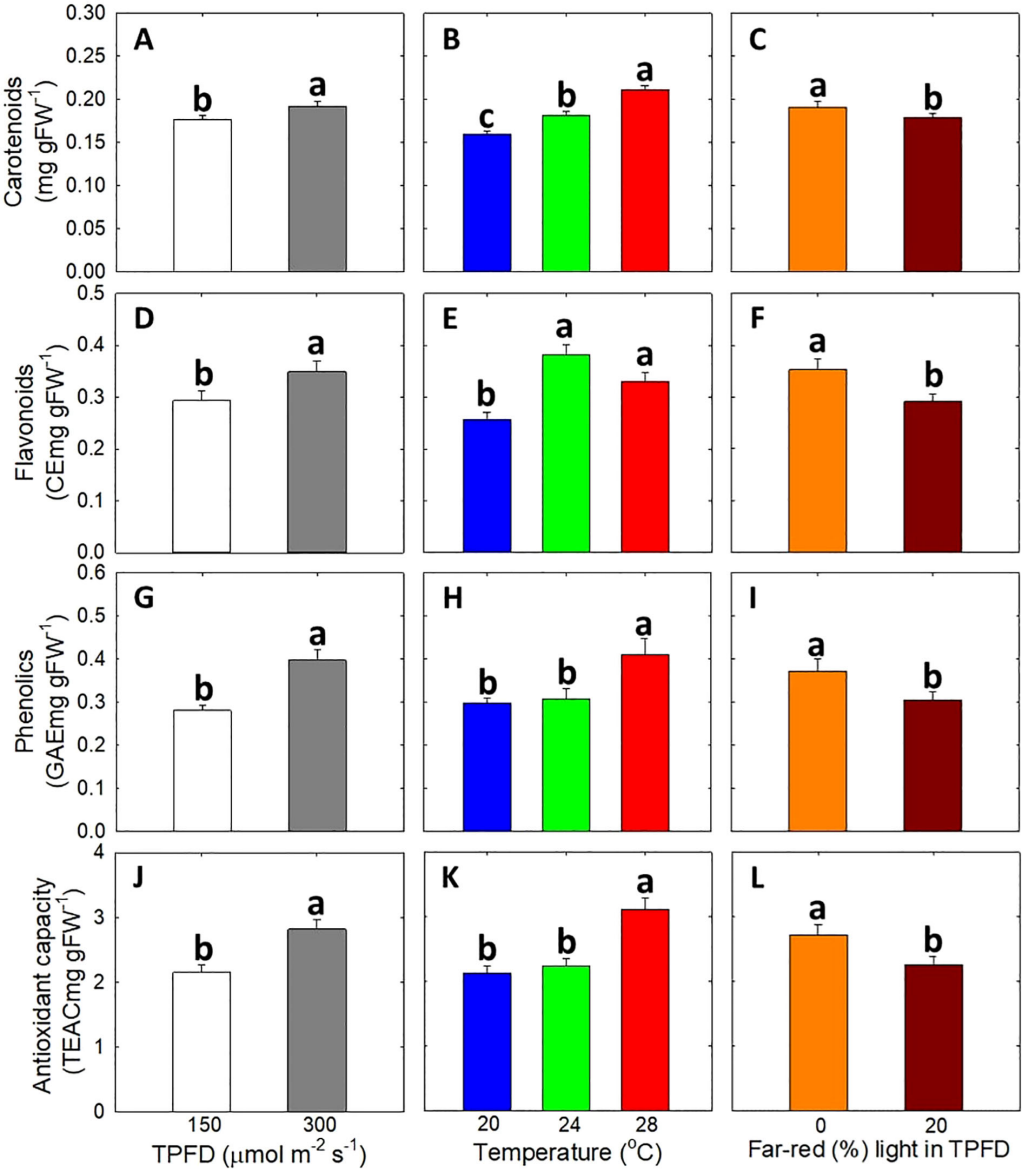
The interactive effect between light spectra and temperature under two light intensities [150 and 300 μmol m^-2^ s^-1^ in total photon flux density (TPFD; 400-800 nm)] on the contents of carotenoids **(A–C)**, flavonoids **(D–F)**, phenolics **(G–I)**, and antioxidant capacity **(J–L)**. No significant interactions among TPFD, temperature, and FR light were observed in these parameters. The two light spectra are denoted based on the percentage of far-red photons (FR; 700-800 nm) in TPFD, i.e., 0 and 20% FR light. The three temperatures were 20, 24, and 28°C. Different letters following the mean ± SE (n = 3 from the 2^nd^ replicate study) indicate significance at *p*< 0.05.

For secondary metabolite contents and antioxidant capacity, there were no significant interactions among TPFD, temperature, and FR light (**Figure 9**). Both higher TPFD and warm temperature of 28°C significantly increased the concentrations of carotenoids, flavonoids, phenolics, and antioxidant capacity, while FR light caused decreases in these parameters. Specifically, higher TPFD enhanced the concentration of carotenoids by 8%, flavonoids by 19%, phenolics by 42%, and antioxidant capacity by 31% (**Figures 9A, D, G, J**). Warm temperature (28°C) also increased the concentration of carotenoids by 32%, flavonoids by 49%, phenolics by 38%, and antioxidant capacity by 46% compared to a cool temperature of 24°C (**Figures 9B, E, H, K**). In contrast, FR light significantly reduced the concentration of carotenoids by 6%, flavonoids by 17%, phenolics by 18%, and antioxidant capacity by 17% (**Figures 9C, F, I, L**).

## 4 Discussion

### 4.1 Interactive effect between FR light and temperature on plant morphology diminished at high light intensity

Previous studies have found that FR light and warm temperature synergistically promote hypocotyl elongation in Arabidopsis seedlings (Patel et al., 2013; Romero-Montepaone et al., 2020; Burko et al., 2022). However, the interactive effect between FR light and warm temperature on leaf expansion, which is crucial for photon capture and crop yield, showed opposite trends compared to hypocotyl elongation (Patel et al., 2013; Jeong et al., 2024). For example, in lettuce, FR light promoted the leaf expansion at cooler temperatures (20°C and 24°C) but not at warmer temperature of 28°C (Jeong et al., 2024). Similarly, we also found that FR light combined with warm temperature (28°C) synergistically enhanced stem elongation, while reducing leaf expansion of lettuce at TPFD_150_ (**Figures 4A, B** and **Supplementary Figure 3**). This indicates a trade-off between leaf expansion and stem elongation. Consequently, a negative correlation between total leaf area and stem length was observed at TPFD_150_, particularly due to the excessive stem elongation under 20% FR light and warmer temperatures (24 and 28°C) (**Figure 5A**). The pronounced stem elongation induced by a combination of FR light and warm temperature (28°C) is likely an evolutionary adaptation to cope with heightened respiratory demands under elevated temperatures (**Figures 7I, K**) (Legris et al., 2017; Romero-Montepaone et al., 2021; Jeong et al., 2024). Such a strategy enables plants to escape shade by extending their stems, strategically positioning their leaves to gain better access to unfiltered sunlight for photosynthesis.

However, when light intensity increased from 150 to 300 μmol m^-2^ s^-1^, the magnitude of the interactive effect between FR light and temperature on leaf expansion and stem elongation diminished, with FR light consistently increasing total leaf area regardless of temperature (**Figures 4A, B**). Unlike the negative correlation between total leaf area and stem length observed at TPFD_150_, a positive correlation between these two parameters was observed at TPFD_300_ (**Figure 5**). These findings consistently indicate that the interactive effects between FR light and warm temperature on plant morphology diminished as light intensity increased (**Figures 4**, **5**, and **Supplementary Figure 1**). The notion is further supported by root morphological data, where the interactive effects between FR light and temperature on total root length and average root diameter disappeared when light intensity increased from 150 to 300 μmol m^-2^ s^-1^ (**Supplementary Figure 2**).

The diminished interactive effect between FR light and warm temperature on plant morphology at high light intensity could be attributed to the reduced effect of thermal reversion on PHY dynamics in two possible ways. Firstly, photoconversion rate increases with increasing light intensity (Sager et al., 1988; Mancinelli, 1988; Klose et al., 2015; Legris et al., 2016). Specifically, the photoconversion rates (both from Pr to Pfr and from Pfr to Pr) increased proportionally with increasing light intensity (Equations 2, 3; Sager et al., 1988; Mancinelli, 1988). However, thermal reversion rates (i.e., k_r1_ and k_r2_) are not influenced by light intensity (Jung et al., 2016; Klose et al., 2015; Legris et al., 2016; Sellaro et al., 2019). Thus, the effect of thermal reversion on the steady-state of PHYB could be overridden by the accelerated photoconversion rates under high light intensity, resulting in a diminished effect of temperature on PHYB activity and PHYB-mediated plant morphological responses (Klose et al., 2015; Sellaro et al., 2019). Secondly, high light intensity can decrease thermal reversion rate by stabilizing active PHYB through nuclear body formation. Specifically, the rate of thermal reversion of PHYB from D_1_ to D_0_ (k_r1_) is significantly faster than those from D_2_ to D_1_ (k_r2_), making the amount of P_fr_-P_r_ heterodimer (D_1_) a crucial determinant for thermal reversion rate (Klose et al., 2015). Previous studies have reported that high light intensity stimulates nuclear body association and increase the size of nuclear bodies, which serve as the storage site stabilizing and protecting PHYB from inactivation (Chen et al., 2003; Rausenberger et al., 2010; Trupkin et al., 2014; Van Buskirk et al., 2014; Legris et al., 2016; Sellaro et al., 2019). Given that P_fr_-P_r_ (D_1_) heterodimers are primarily located in nucleoplasm rather than nuclear body (Klose et al., 2015; Sellaro et al., 2019), a larger nuclear body size at high light intensity indicates a higher proportion of D_2_ and a smaller D_1_ pool. Therefore, the nuclear body association at high light intensity reduces the size of the D_1_ pool and, consequently, reduces thermal reversion (Trupkin et al., 2014; Klose et al., 2015; Legris et al., 2016; Sellaro et al., 2019). Consistently, Sellaro et al. (2019) found that higher light intensities were necessary to reach PPE under warmer temperatures, suggesting that thermal reversion would have a bigger impact on phytochrome dynamics and plant morphology under warm temperatures and low light intensities. In this study, a significant interaction between the estimated PPE and temperature was observed in leaf expansion rate and stem elongation rate under a lower light intensity of 150 μmol m^-2^ s^-1^ (**Supplementary Figure 5**). Specifically, leaf expansion rate decreased as the estimated PPE increased (i.e., when FR light was absent) at 20°C (α = -35.6), but increased as the estimated PPE increased at 24°C (α = 122.9) and 28°C (α = 173.2) (**Supplementary Figure 5A**). The effect of PPE on stem elongation rate became more pronounced as temperature increased from 20°C to 28°C (α = -0.22 at 20°C, -1.01 at 24°C, and -2.24 at 28°C) (**Supplementary Figure 5C**). In contrast, at high light intensity, the effect of PPE on leaf expansion rate and stem elongation rate were similar across the three temperatures, and there were no significant interactions between temperature and the estimated PPE (**Supplementary Figures 5B, D**). Taken together, our results, along with previous findings, suggest that at lower light intensity, light spectra and temperature interactively regulate the steady-state of PHYB and plant morphology. However, at high light intensity, PHYB activity and its effect on plant morphology are likely predominantly regulated by light spectra and are less affected by temperature.

### 4.2 FR light consistently enhanced biomass accumulation at higher light intensity

Photon capture and plant biomass typically exhibit a strong linear correlation (Monteith, 1977; Gifford et al., 1984). Consistently, we found a strong positive correlation between total DW and total intercepted photons (R^2^ = 0.94) (**Figure 8A**). Therefore, enhancing leaf expansion and photon capture through the application of FR light can be an effective strategy to increase plant biomass in indoor vertical farming systems (Park and Runkle, 2017; Meng and Runkle, 2019; Zhen and Bugbee, 2020; Legendre and van Iersel, 2021). However, our results showed that the effects of FR light on leaf expansion, canopy photon capture, and biomass accumulation were dependent on temperature and light intensity (**Figures 4**, **6**, and **Supplementary Figure 3**). Specifically, FR light did not affect total DW at 20°C but caused reductions in plant biomass at warmer temperatures (24°C and 28°C) under low light intensity, while consistently increasing biomass accumulation across all temperature conditions at high light intensity (**Figure 6A**). The excessive stem growth induced by FR light and warmer temperature (24-28°C) at low light intensity (TPFD_150_) led to decreased leaf area and, consequently, reduced photon capture and biomass production (**Figure 5**). These results underscore the importance of preventing excessive stem growth via providing adequate light intensity when utilizing FR light to enhance leaf expansion and plant growth under warm temperature (**Figure 5**).

We also found that total DW was only weakly correlated with the quantum yield of PSII and leaf-level photosynthetic rate (R^2^ = 0.17 for *Φ_PSII_* and R^2^ = 0.24 for *P_net_*), compared to total intercepted photons (R^2^ = 0.94) (**Figure 8**). These results indicate that plant biomass accumulation is more dependent on canopy photon capture, compared to the single-leaf photosynthetic efficiency. Consistently, previous studies also reported that the photosynthetic activities at single-leaf level oftentimes do not predict crop yield (Evans, 1975; Elmore, 1980).

### 4.3 The combination of high light intensity, warm temperature, and FR light maximized both lettuce yield and health-promoting compounds

Enhancing health-promoting compounds, including photosynthetic pigments and secondary metabolites, is an important production goal in indoor farming systems (Poiroux-Gonord et al., 2010; Yang et al., 2018). Previous research has found that the FR light generally reduces the contents of photosynthetic pigments and secondary metabolites, resulting in lower nutritional quality (Li and Kubota, 2009; Stutte et al., 2009; Kong and Nemali, 2021). Similarly, we found that FR light decreased the contents of various phytochemicals, including chlorophyll a, carotenoids, flavonoids, and phenolics (**Figure 9** and **Supplementary Figure 4**). The reduced phytochemicals under FR light may be at least partially due to its direct effect on down-regulating the expression of genes involved in the biosynthetic pathways of these phytochemicals through the PHY signaling networks (Huq et al., 2004; Toledo-Ortiz et al., 2010). The reduction in phytochemical contents may also be attributed to the dilution effect due to increased leaf expansion induced by FR light (Casal et al., 1987; Li and Kubota, 2009; Zhen and Bugbee, 2020; Kong and Nemali, 2021).

In contrast to the responses under FR light, higher light intensity improved the levels of secondary metabolites and antioxidant capacity (**Figure 9**). Similar results were also observed in several crop species (Tattini et al., 2014; Dou et al., 2018; Pérez-López et al., 2018). The increased concentration of phytochemicals at higher light intensity may be attributed to the increased availability of photosynthates (**Figure 7**) (Mosaleeyanon et al., 2005). Similarly, warmer temperature also increased the levels of those antioxidants (**Figure 9** and **Supplementary Figure 4**) (Lefsrud et al., 2005; Shamloo et al., 2017). This enhancement of phytochemicals and antioxidant capacity may be due to the protective mechanism against increased oxidative stress under warm temperature of 28°C (Oh et al., 2009; Pospíšil, 2016). However, in this study, warm temperature of 28°C did not cause significant stress as plants grown under 28°C showed high *F_v_*/*F_m_* values between 0.81-0.82 (Maxwell and Johnson, 2000; Baker, 2008).

We found that the reductions in health-promoting compounds and antioxidant capacity induced by FR light could be compensated for by increasing light intensity and temperature. The treatment with higher light intensity, warmer temperature, and 20% FR light (i.e., TPFD_300_ x 28°C x 20% FR light) increased crop yield [shoot (leaf + stem) FW] by 400% and antioxidant capacity by 60%, compared to the treatment with lower light intensity, cool temperature, and 0% FR light (i.e., TPFD_150_ x 20°C x 20% FR light). This result indicates that the combination of high light intensity, warm temperature, and FR light can maximize not only plant biomass but also nutritional quality.

## 5 Concluding remarks

At lower light intensity, FR light and temperature interactively regulated lettuce growth and morphology. Specifically, at TPFD of 150 μmol m^-2^ s^-1^, FR light enhanced leaf expansion only at a cool temperature of 20°C. However, at TPFD of 300 μmol m^-2^ s^-1^, the interactive effect of FR light and warm temperature diminished, leading to a consistent stimulative effect of FR light on canopy photon capture, consequently enhancing plant biomass at all three temperatures (20, 24 and 28°C). Plant biomass was primarily dependent on canopy photon capture, rather than the photosynthetic efficiency at the single-leaf level. Furthermore, light intensity and warm temperature enhanced the concentrations of chlorophylls, carotenoids, flavonoids, and phenolics, as well as the antioxidant capacity, while FR light reduced these parameters. Overall, we found that the combination of high light intensity, warm temperature, and FR light resulted in the highest plant yield and antioxidant capacity. This interdependent relationship among environmental factors and their influence on plant biomass and quality underscores the importance of co-optimizing multiple environmental factors to maximize crop yield and nutritional quality in a controlled environment production system.

## Data Availability

The raw data supporting the conclusions of this article will be made available by the authors, without undue reservation.
