# Supplementary material for: The interactive effects between far-red light and temperature on lettuce growth and morphology diminish at high light intensity
Source: Front Plant Sci. 2024 Dec 2;15:1497672. doi: 10.3389/fpls.2024.1497672 (PMC11646736; doi:10.3389/fpls.2024.1497672)
Supplement: Supplementary file 1 [file DataSheet1.docx]

Supplementary Material

The Interactive Effects between Far-red Light and Temperature on Lettuce Growth and Morphology Diminish at High Light Intensity

**Sang Jun Jeong^1,2^, Qianwen Zhang^2,3^, Genhua Niu^2*^, and Shuyang Zhen^1*^**

*** Correspondence:**

Shuyang Zhen: [shuyang.zhen@ag.tamu.edu](mailto:shuyang.zhen@ag.tamu.edu); Genhua Niu: [genhua.niu@ag.tamu.edu](mailto:genhua.niu@ag.tamu.edu)

## Supplementary Figures


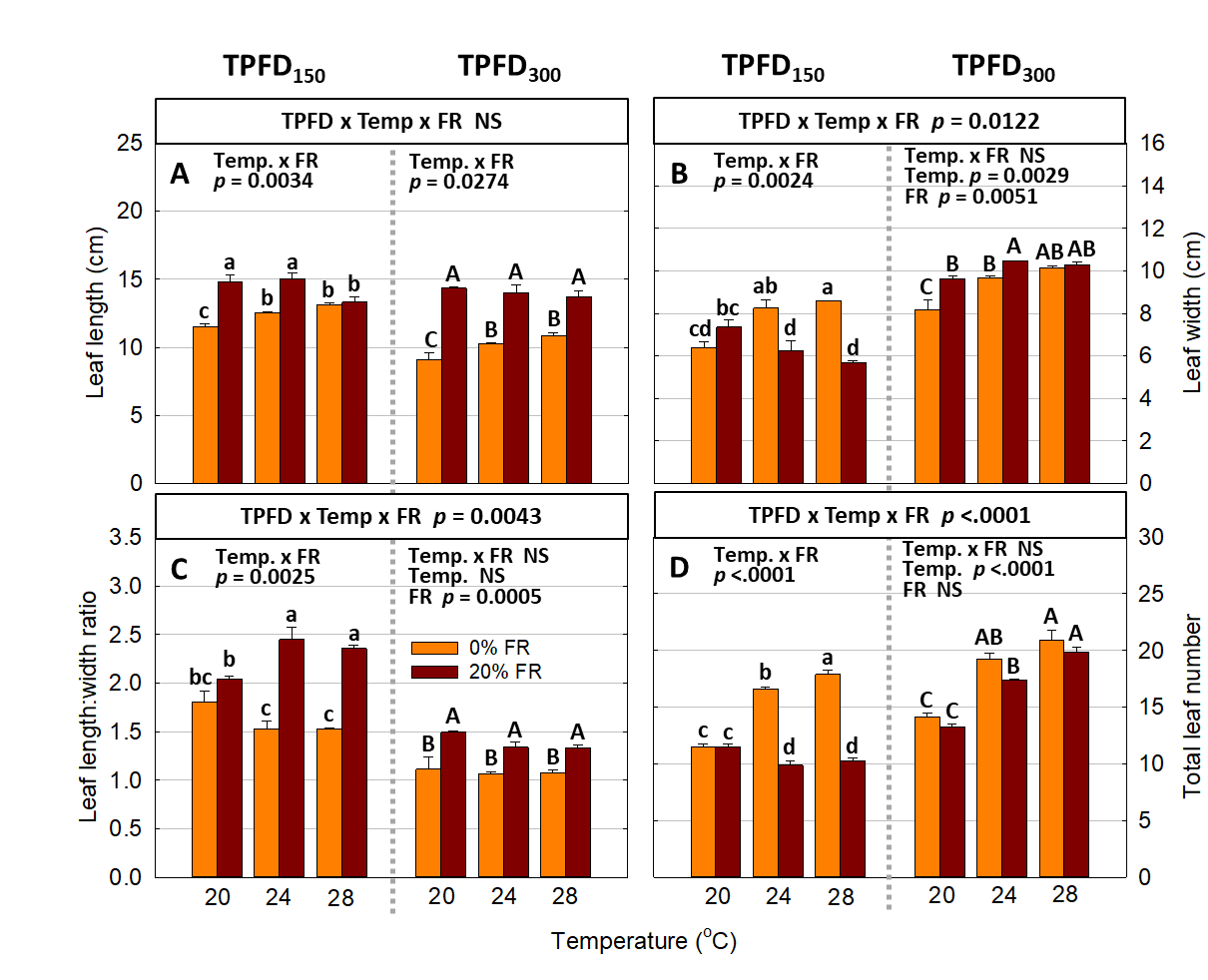


**Supplementary Figure 1.** The interactive effect between light spectra and temperature under two light intensities [150 and 300 μmol m^-2^ s^-1^ in total photon flux density (TPFD; 400-800 nm)] on leaf length (A), leaf width (B), leaf length:width ratio (C), and total leaf number (D). The two light spectra are denoted based on the percentage of far-red photons (FR; 700-800 nm) in TPFD, i.e., 0 and 20% FR light. The three temperatures were 20, 24, and 28 ℃. Different letters following the mean ± SE [n = 2; subsamples (4 plants per treatment per replicate study) were averaged before statistical analysis] indicate significant difference among the six treatments (three temperatures x two FR levels) at each light intensity at *p* < 0.05. NS stands for non-significance.


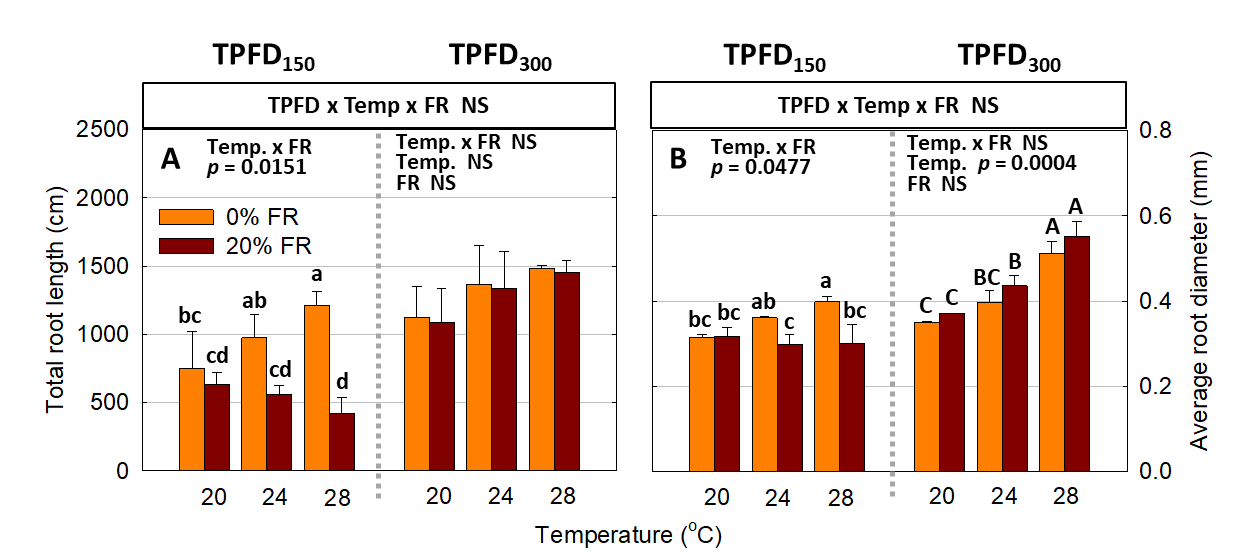


**Supplementary Figure 2.** The interactive effect between light spectra and temperature under two light intensities [150 and 300 μmol m^-2^ s^-1^ in total photon flux density (TPFD; 400-800 nm)] on total root length (A) and average root diameter (B). The two light spectra are denoted based on the percentage of far-red photons (FR; 700-800 nm) in TPFD, i.e., 0 and 20% FR light. The three temperatures were 20, 24, and 28 ℃. Different letters following the mean ± SE [n = 2; subsamples (4 plants per treatment per replicate study) were averaged before statistical analysis] indicate significant difference among the six treatments (three temperatures x two FR levels) at each light intensity at *p* < 0.05. NS stands for non-significance.


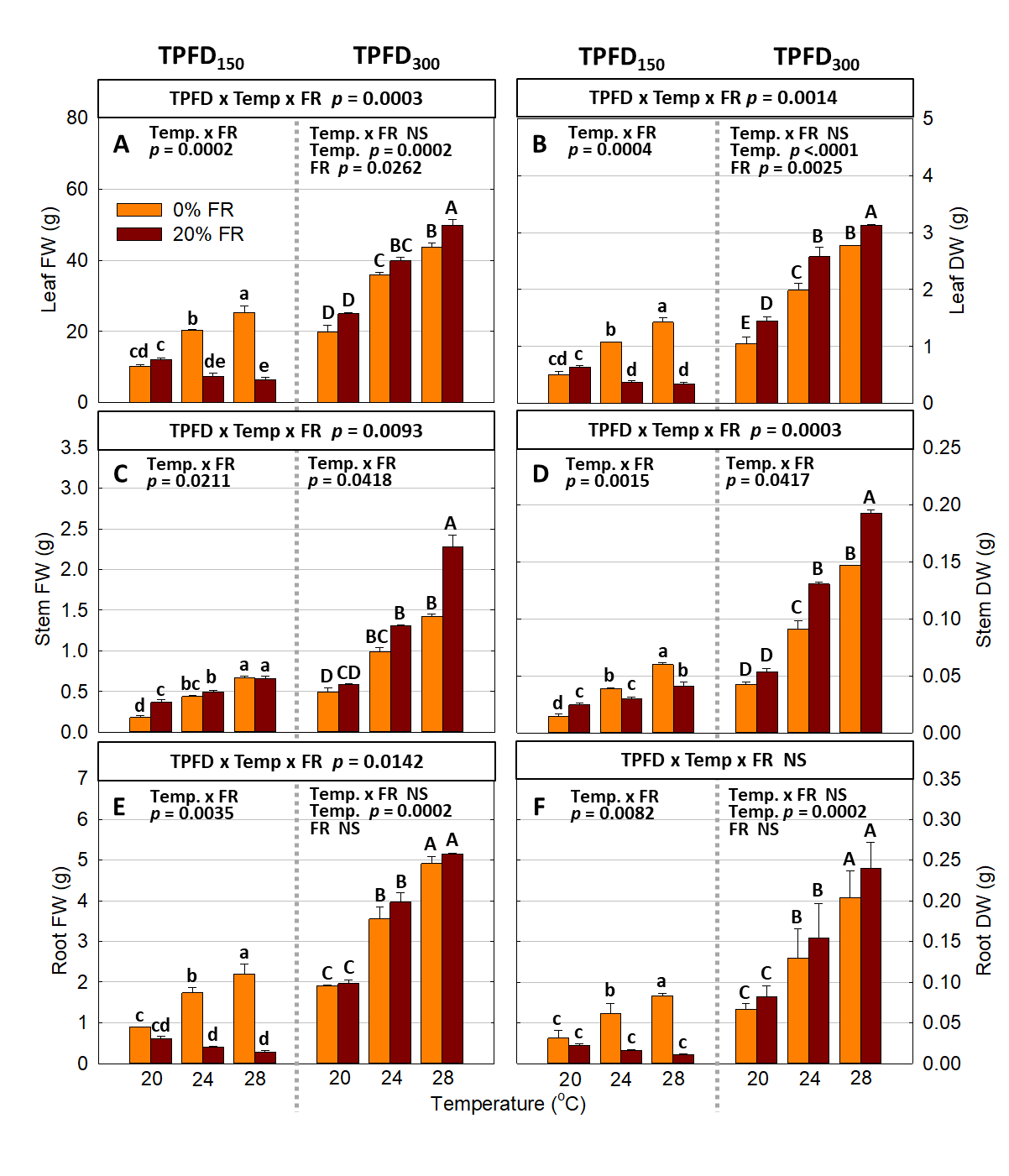


**Supplementary Figure 3.** The interactive effect between light spectra and temperature under two light intensities [150 and 300 μmol m^-2^ s^-1^ in total photon flux density (TPFD; 400-800 nm)] on leaf fresh weight (FW) (A), leaf dry weight (DW) (B), stem FW (C), stem DW (D), root FW (E), and root DW (F). The two light spectra are denoted based on the percentage of far-red photons (FR; 700-800 nm) in TPFD, i.e., 0 and 20% FR light. The three temperatures were 20, 24, and 28 ℃. Different letters following the mean ± SE [n = 2; subsamples (4 plants per treatment per replicate study) were averaged before statistical analysis] indicate significant difference among the six treatments (three temperatures x two FR levels) at each light intensity at *p* < 0.05. NS stands for non-significance.


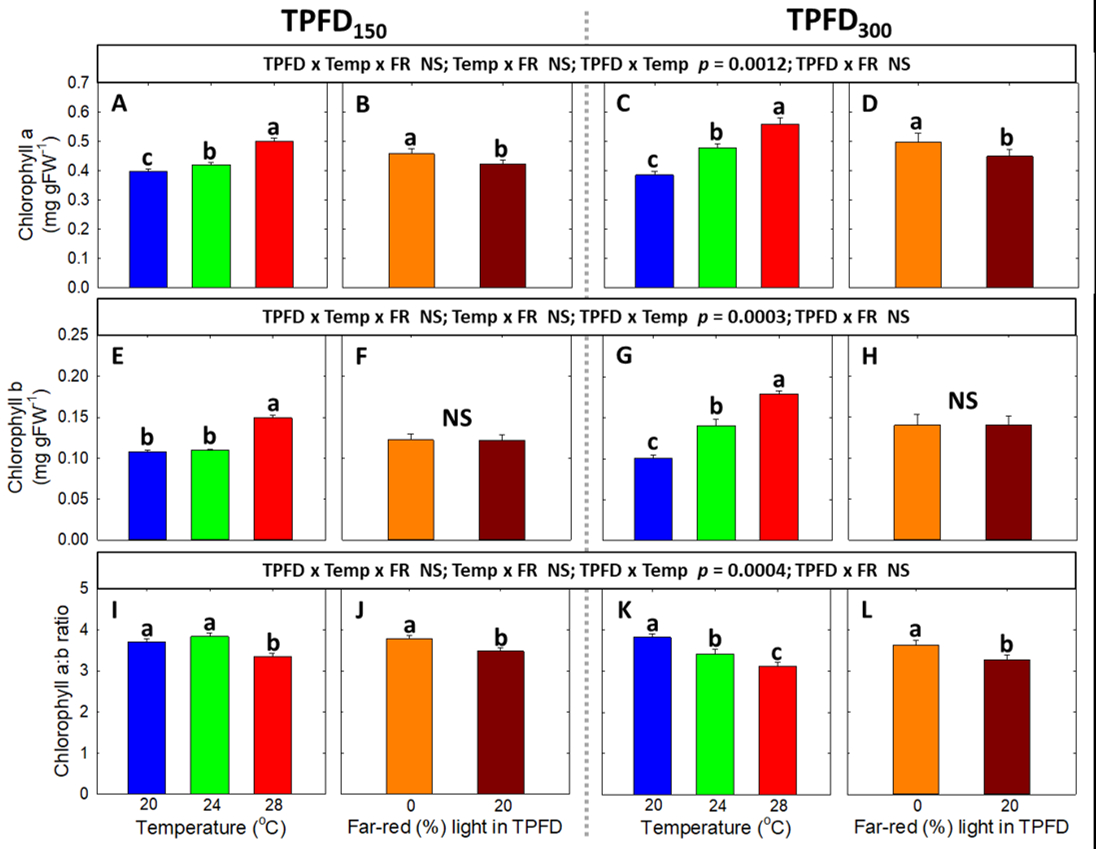


**Supplementary Figure 4.** The interactive effect between light spectra and temperature under two light intensities [150 and 300 μmol m^-2^ s^-1^ in total photon flux density (TPFD; 400-800 nm)] on the contents of chlorophyll a (A-D), chlorophyll b (E-H), and chlorophyll a:b ratio (I-L). The two light spectra are denoted based on the percentage of far-red photons (FR; 700-800 nm) in TPFD, i.e., 0 and 20% FR light. The three temperatures were 20, 24, and 28 ℃. Different letters following the mean ± SE (n = 3 from the 2^nd^ replicate study) indicate significance at *p* < 0.05. NS stands for non-significance.


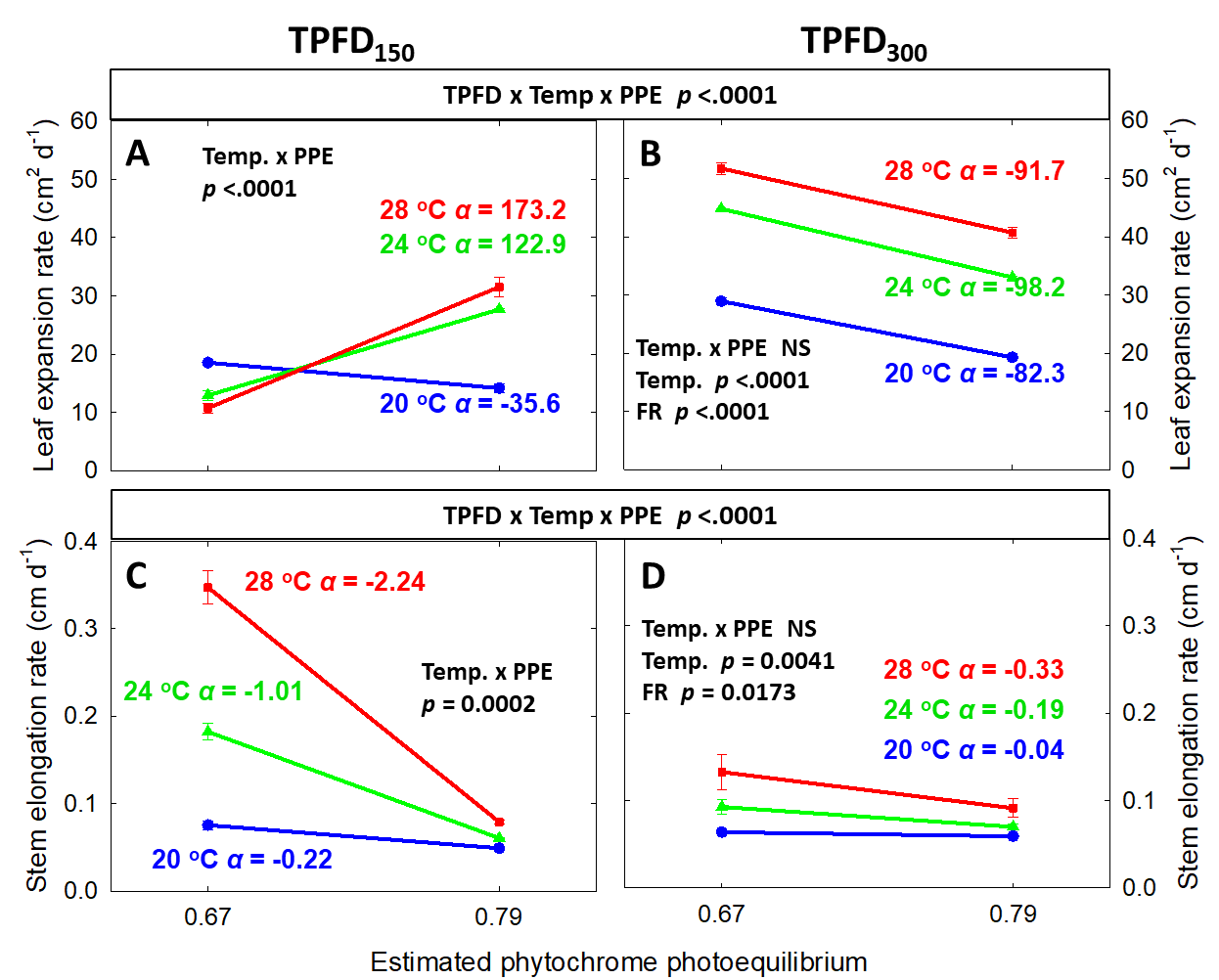


**Supplementary Figure 5.** Interactions between the estimated phytochrome photoequilibrium (PPE) and temperature under two light intensities. PPE was estimated based on the simplified three-state model with the assumption that thermal reversion rates were zero (i.e., k_r1_ = k_r2_ = 0) (Sellaro et al., 2019). Correlation between PPE and leaf elongation rate (A and B) and stem elongation rate (C and D) were examined under three temperatures (20, 24, and 28 ℃) and two light intensities [150 μmol m^-2^ s^-1^ (TPFD_150_) and 300 μmol·m^-2^·s^-1^ (TPFD_300_)]. Each data point represents mean ± SE [n = 2; subsamples (4 plants per treatment per replicate study) were averaged before statistical analysis]. NS stands for non-significance. The slope (*α*) for each line graph is presented to compare the correlations between the estimated PPE and plant morphological parameters under different temperatures and light intensities.

## Supplementary Tables

**Supplementary Table 1.** Light intensity, temperature, and light spectral characteristics of twelve treatments (two light intensities x three temperatures x two light spectra). Light spectra consisted of blue (B; 400-500 nm), green (G; 500-600 nm), red (R; 600-700 nm), and far-red (FR; 700-800 nm) photons from light-emitting diodes. The subscript after each waveband indicates its photon flux density in μmol m^-2^ s^-1^.

| Light intensity^z^  (μmol m^-2^ s^-1^) | Temperature  (℃) | Light spectra | %FR in TPFD^x^ | Actual TPFD  ± SD^w^ | Estimated PPE^v^ |  |  |
| --- | --- | --- | --- | --- | --- | --- | --- |
| 150 | 20 | B_15_ + G_15_ + R_120_ | 0 | 152.9 ± 8.5 | 0.79 |  |  |
|  |  | B_15_ + G_15_ + R_90_ + FR_30_ | 20 | 154.0 ± 7.1 | 0.67 |  |  |
|  | 24 | B_15_ + G_15_ + R_120_ | 0 | 151.1 ± 10.4 | 0.79 |  |  |
|  |  | B_15_ + G_15_ + R_90_ + FR_30_ | 20 | 149.1 ± 10.1 | 0.67 |  |  |
|  | 28 | B_15_ + G_15_ + R_120_ | 0 | 152.8 ± 6.7 | 0.79 |  |  |
|  |  | B_15_ + G_15_ + R _90_ + FR_30_ | 20 | 149.4 ± 10.5 | 0.67 |  |  |
| 300 | 20 | B_30_ + G_30_ + R_240_ | 0 | 301.3 ± 15.9 | 0.79 |  |  |
|  |  | B_30_ + G_30_ + R _180_ + FR_60_ | 20 | 298.5 ± 14.3 | 0.67 |  |  |
|  | 24 | B_30_ + G_30_ + R_240_ | 0 | 302.5 ± 19.0 | 0.79 |  |  |
|  |  | B_30_ + G_30_ + R _180_ + FR_60_ | 20 | 300.6 ± 22.6 | 0.67 |  |  |
|  | 28 | B_30_ + G_30_ + R_240_ | 0 | 302.8 ± 16.7 | 0.79 |  |  |
|  |  | B_30_ + G_30_ + R _180_ + FR_60_ | 20 | 297.8 ± 16.0 | 0.67 |  |  |
| ^z^Light intensity: total photon flux density (TPFD in μmol m^-2^ s^-1^) integrated from 400 to 800 nm.  ^y^%FR in TPFD: percentage of far-red photons (700-800 nm) in total photon flux density (400 to 800 nm).  ^w^SD: Standard deviation  ^v^Estimated PPE: Phytochrome photoequilibrium was estimated based on the simplified three-state model by setting thermal reversion rates to zero (i.e., k_r1_ = k_r2_ = 0), following Sellaro et al. (2019). | | | | | | |  |
